# Clinical solutions: not always what they seem?

**DOI:** 10.1186/s13054-015-0870-4

**Published:** 2015-05-07

**Authors:** Anne Burke-Gaffney, Benedict C Creagh-Brown

**Affiliations:** Vascular Biology, Cardiovascular Sciences, National Heart and Lung Institute Division, Faculty of Medicine, Imperial College London, Dovehouse Street, London, SW3 6LY UK; Intensive Care and Respiratory Medicine, The Royal Surrey County Hospital NHS Foundation Trust, Egerton Road, Guildford, GU2 7XX UK; Surrey Perioperative Anaesthesia and Critical care collaborative Research group (SPACeR), Faculty of Health and Medical Science, Duke of Kent Building, University of Surrey, Guildford, Surrey GU2 7TE UK

Brenner and colleagues [1], in their article published in *Critical Care*, showed elevated levels of the reactive carbonyl species (RCS) methylglyoxal (MG) in the circulation of patients with septic shock. We commend the authors’ bravery in launching this molecule into a field well-populated with biomarkers and where clinical diagnosis persists as the ‘gold standard’ [2].

The authors hypothesised that MG accumulation resulted from metabolic dysregulation and oxidative stress associated with septic shock. Impairment of MG detoxification was also proposed as a contributory factor. However, whether MG was, at least in part, inadvertently administered during routine clinical care appears not to have been considered.

Some clinical solutions, such as peritoneal dialysis fluid, are known to contain MG and other RCS [3,4]. Bearing in mind that patients with septic shock, managed on intensive care units, typically receive large volumes of intravenous fluids and, in due course, enteral (and parenteral) nutrition, could these solutions not also be a source of MG and other RCS? Also, 25% of the septic shock patients in Benner and colleagues’ study [1] had acute liver failure, and such patients often require infusions of high concentration dextrose, a further potential source of RCS. Given the growing awareness that clinical solutions are not always what they seem, we would suggest that measurements of MG and other RCS levels in solutions administered to patients with sepsis could be a helpful and perhaps an illuminative and valuable addition not only to this study but to the sepsis shock/intensive care field in general.

## Abbreviations

MG: Methylglyoxal
RCS: Reactive carbonyl species
